# A highly sensitive lateral flow immunoassay for the rapid and on-site detection of enrofloxacin in milk

**DOI:** 10.3389/fnut.2022.1036826

**Published:** 2022-10-24

**Authors:** Munirah Alhammadi, Jingon Yoo, Sonam Sonwal, So Young Park, Reddicherla Umapathi, Mi-Hwa Oh, Yun Suk Huh

**Affiliations:** ^1^Department of Biological Sciences and Bioengineering, NanoBio High-Tech Materials Research Center, Inha University, Incheon, South Korea; ^2^National Institute of Animal Science, Rural Development Administration, Wanju, South Korea

**Keywords:** biosensor, enrofloxacin analogs, gold nanoparticles, lateral flow immunochromatographic assay (LFIA), rapid diagnosis strips

## Abstract

Enrofloxacin (ENR) is a veterinary antibiotic used to treat bacterial infections in livestock. It chiefly persists in foods and dairy products, which in turn pose severe risks to human health. Hence it is very important to detect the ENR in foods and dairy products to safeguard human health. Herein, we attempted to develop a single-step detection lateral flow immunochromatographic assay (LFIA) using gold nanoparticles (AuNPs) for the rapid and on-site detection of ENR in milk samples. An anti-enrofloxacin monoclonal antibody (ENR-Ab) was conjugated with AuNPs for the specific detection of ENR in milk samples. For sensitivity improvement, many optimization steps were conducted on LFIA test strips. The visual limit of detection (vLOD) was found to be 20 ng/ml with a cut-off value of 50 ng/ml in the milk samples. The obtained LOD and cut-off value were within the safety limit guidelines of the Ministry of food and drug safety, South Korea. The test strip showed negligible cross-reactivity with ENR analogs, and other components of antibiotics, this indicates the high specificity of the LFIA test strip towards ENR. The designed test strip showed good reliability. The visual test results can be seen within 10 min without the need for special equipment. Therefore, the test strip can be employed as a potential detection strategy for the qualitative on-site detection of enrofloxacin in milk samples.

## Graphical Abstract

Rapid on-site detection of enrofloxacin in milk. 
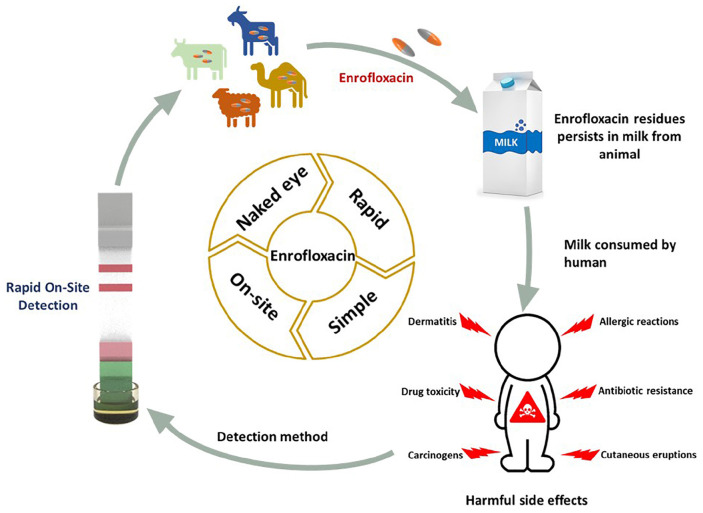

## Introduction

The use of veterinary drugs is necessary for both the prevention and the treatment of various infectious diseases. In general, veterinary drugs can be used for therapeutic, prophylactic, and metaphylactic purposes, or as growth promoters. In animal livestock, infectious disease is a significant factor affecting the profitability of livestock industries (1). However, it is strictly advisable to carefully use these veterinary drugs for animal and human consumption. Inappropriate and uncontrolled usage of these drugs results in causing the potential risks associated with microbial resistance, which ultimately influences the animal production system and also leads to toxicological effects on human health and the surrounding environment. The intake of these veterinary drugs above the safety limits will leads to chronic allergies and human intestinal flora (2).

Fluoroquinolones are the class of antibiotics that are widely used in veterinary and human medicine (3, 4). Enrofloxacin is one antibiotic that comes under the family fluoroquinolones. Nowadays enrofloxacin (ENR) is widely used as an antibacterial agent in the aquaculture industry, poultry, dairy industry, and livestock husbandry. ENR has a broad-spectrum antibacterial activity that works as an inhibitor of enzymes which control DNA topology; topoisomerase and gyrase (5, 6). The wide use of ENR as veterinary medicine in poultry and dairy, poses severe health-related issues and risks to humans, as humans are consuming dairy and poultry products frequently. ENR residues can be commonly seen in dairy and poultry products. Injecting ENR into the lactating breeding animals can leave traces of ENR residues in the meat tissues and dairy milk. If humans will consume this meat and milk, it will become a potential hazard and causes allergic reactions. On the other hand, it was reported that owing to the existence of ENR residues in the foods obtained from dairy and poultry products, the efficiency of ENR potentiality in humans is decreasing.

To safeguard public wellness against the veterinary drugs in the meat and dairy products from animal sources, maximum residue limits (MRLs) regulations were instituted by the ministry of food and drug safety (MFDS), South Korea. In Korea, the MRLs for veterinary drugs and their associated metabolites were based on the various categories of drug residue depletion investigations (7). If the concentration of drug residue in the food or dairy products is in the below tolerance range of MRL, then it is considered safe food which can be consumed daily. In contrast, if it is above the tolerable range of MRL, then it is considered toxic food (8). This specifies the need for widespread and effective regulations to control the ENR content in milk and other foodstuffs. Hence it is of utmost importance to regularly check the ENR residues in the foods and dairy products of animal origin (9). According to MFDS, South Korean guidelines, the MRLs for ENR in milk samples should be 0.05 mg/kg (10). In this regard developing simple, low-cost, labor free and rapid analytical tools are mandatory.

Many conventional techniques have been used for the detection of ENR in food. For instance, Terrado-Campos et al. detected ENR in porcine and bovine meat using high-performance liquid chromatography (11). Other conventional techniques such as capillary electrophoresis (12), mass spectrometry (13, 14), surface plasmon resonance (15), surface-enhanced Raman scattering (16), and matrix solid-phase dispersion (17). Although these tools possess high sensitivity, selectivity, and appreciable accuracy, still, they require ultrafine chemicals, sophisticated maintenance, and professional and well-trained technicians. Moreover, the experimental protocols are time-consuming, complex sample pretreatment procedures. All these disadvantages make it impossible for the on-site detection of ENR residues. Recently, diverse systems and methods with detection advances compared to conventional have been developed for more rapid, easy, and sensitive detection of ENR. For instance, Li et al. (18) developed a molecularly imprinted polymers-chemiluminescence system for the determination of ENR in milk. Aymard et al. (19) fabricated a novel electrochemical immunosensor for the detection of ENR in meat. Photoelectrochemical sensors (20) have been developed for the detection of ENR in milk. Recently, Huang et al. (21) extensively reviewed the sandwich, adsorption-desorption, and competitive aptamer-based LFAs for the detection of various target analytes. Further, multiplexed detections and signal amplification strategies were discussed to deliver an overview of the aptamer-based LFAs for the detection of various target analytes. LFIA possesses several advantages such as being cost-effective, being independent of the cold chain, easy handling, real-time feedback of results, rapid diagnosis strips, and point-of-care testing (POCT) (22–25). Antibodies and labeling materials are the basis for immunological sensing strategies (26–28). Various types of signal transducers such as colloidal gold nanoparticles (AuNPs) (29–31), quantum dots (32), and polymer microspheres (33) have been employed in the immunological assays for the detection of ENR in foods. Lateral flow immunochromatographic assay (LFIA) has been widely employed as a paper-based biosensor for the on-site detection of ENR residues. LFIA can efficiently screen the target analytes in a given sample and attain the results in a few minutes without the requirement of a well-trained operator, or expensive and sophisticated experimental tools (21).

Among the various approaches, AuNP-based LFIA has attained widespread attention and it developed as an emerging technology owing to its eye-reading results, long-term stability, free of complex instruments, and easy operation procedure. It utilizes low-cost and easily synthesizable colloidal AuNPs, as reporters in the immunoreactions (34). Moreover, AuNPs display little biological toxicity and excellent biocompatibility, which makes them advantageous in the *in vivo* and *in vitro* systems. Hence, the point-of-care technology strip based on AuNPs-LFIA can work as a rapid, low-cost, and efficient tool for the on-site detection of hazardous components. Until now, the AuNPs- LFIA have been widely used to detect various analytes such as infectious viruses, drugs, proteins, and pathogens (9, 22, 35). Studies on the AuNPs-based LFIA for the detection of antibacterial agents in the food samples are very less. Therefore, in this study, we focused on the development of a portable, rapid, highly sensitive, and specific LFIA sensor based on a commercial specific monoclonal antibody conjugated AuNPs for the on-site detection of ENR in milk samples. The developed approach can be easily performed by non-professional personnel, as the proceeded milk sample tests can be executed without any sample pretreatment. The obtained LOD and cut-off value were within the safety limit guidelines of MFDS, South Korea.

## Materials and methods

### Materials

Enrofloxacin, ciprofloxacin, ofloxacin, norfloxacin, tetracycline, creatine monohydrate, bovine serum albumin (BSA), polyvinyl alcohol (PVA), Tween 20, and Gold (III) chloride trihydrate (HAuCl4.3H2O, ≥99.9) were purchased from Sigma–Aldrich (St. Louis, MO, USA). Ampicillin sodium and chloramphenicol were purchased from Duchefa Biochemie (Haarlem, Netherlands). Trisodium citrate dihydrate was purchased from Kanto Chemical Co., Inc. (Tokyo, Japan). Anti-enrofloxacin monoclonal antibody (ENR-Ab) produced in mice and coating antigen BSA-ENR were purchased from Creative Diagnostics, (New York, USA). The backing card, absorbent pad, sample pad, nitrocellulose membrane, and conjugation pad used to fabricate LFIA strips, were purchased from Bore Da Biotech Co., Ltd. (Seongnam, Korea). Milk samples were purchased from a local market in Incheon, South Korea. All other reagents and solvents used were of analytical grade or higher.

### Methods and instruments

Distilled water was obtained from Millipore (Molsheim, France). Cutter AR-100CG (Namyangju, Korea) was used for cutting the sheets. Rotator HAG (Gunpo, Korea) was used for rotating the samples. Centrifuge (VS-15000CFNII) (Daejeon, Korea) was used for the centrifugation of the samples. UV-vis spectra and plasmon absorbance peak of the samples were examined using V-770 UV-Visible/NIR Spectrophotometer (TS Science, Seoul, Korea). The size and morphology of the synthesized gold nanoparticles (AuNPs) were analyzed using field-emission transmission electron microscopy (FE-TEM; JEM2100F, JEOL Ltd., USA).

### Synthesis of colloidal gold nanoparticles

AuNPs with 35 nm size were synthesized by following the previously reported method (36) with minor modifications following different conditions in the laboratory. For the synthesis of AuNPs with 35 nm, 200 ml of deionized water is added to a flat-bottom three-neck reaction vessel, heated to 380C, and stirred at 200 rpm for 10 min. Later, 10% HAuCl_4_.3H_2_O (700 μl) aqueous solution was added. After 2 min, 1% trisodium citrate solution was added and stirred for 15-20 min. Finally, the solution will change from yellow to wine color. Potassium carbonate (K_2_CO_3_) solution was used to adjust the pH to 9.0. of the AuNPs. The synthesized AuNPs were used to fabricate LFIA strips.

### Lateral flow immunochromatographic assay strip optimization

For the development of a highly sensitive LFIA strips for the detection of ENR, an optimization process of different parameters was conducted. We optimized ENR-Ab concentration conjugated to AuNPs (0, 0.5, 1.0, 1.5 μg/ml), blocking buffers (10 mM phosphate buffer (PB), 10 mM phosphate-buffered saline (PBS), and 20 mM borate buffer), blocking buffer pH (7, 8, and 9) and running buffers which includes tween 20 and NaCl with different concentrations and then analyzed the results to improve the assay sensitivity.

### Fabrication of lateral flow immunochromatographic assay strips for enrofloxacin detection

A total of 5.0 ml of AuNPs with optical density (OD) 1 at 526.7 nm wavelength solution was added to different concentrations (0, 0.5, 1.0, 1.5 μg/ml) of ENR-Ab and reacted in a rotator at room temperature for 30 min. Herein, we followed a direct coupling of ENR-Ab to the AuNPs by passive adsorption referring to reported literature (33, 37, 38). This method is commonly used in the conjugation of Ab to AuNPs due to its simplicity, rapidity, and cost-efficiency. The ENR-Ab is freely adsorbed to the surface of the naked AuNPs via electrostatic absorption. After that, AuNPs- ENR-Ab solution was centrifuged (6,000 rpm, 30 min, 4°C) to remove the non-reacted antibodies. 1% of BSA was added, for the further resuspension of the solution. 20 mM borate buffer with pH 9 was used as a blocking buffer to prevent non-specific binding and the solution was reacted for another 30 mins using a rotator. Further, the solution was centrifuged (10,000 rpm, 15 min, 4°C) and removed the supernatant. Blocking and centrifugation steps were repeated twice, to remove the impurities. Finally, ENR-Ab conjugated AuNPs precipitate was resuspended using 1% BSA, 5% sucrose, and borate buffer, and the solution was tuned to an optimized OD ∼8. Conjugation of various concentrations of ENR-Ab with AuNPs is confirmed by using UV-vis spectroscopy, by observing the differences in absorbance peaks as referred to in the literature (39, 40) and by the visual assessment confirmation as shown in Figure 3.

Lateral flow immunochromatographic assay test strips are composed of a conjugation pad (7 mm × 300 mm), absorbent pad (16 mm × 300 mm), sample pad (17 mm × 300 mm), and nitrocellulose membrane (NC membrane) were assembled on a plastic adhesive backing layer (41). The solution containing the target analyte is dropped on the sample pad. The sample pad facilitates the even distribution of the sample and delivers the sample to the conjugate pad (42). A conjugate pad composed of glass fibers was pretreated with 0.05% polyvinyl alcohol (PVA) and 0.05% Tween 20 for 12 h and then dried overnight to prevent the non-specific binding. ENR-Ab conjugated AuNPs solution was added dropwise on the conjugate pad and dried overnight at room temperature in the desiccator. NC membrane contain immobilized test and control lines which were dispersed each with 1.0 mg/ml concentration over it by a dispenser. The test line contains immobilized coating antigen (BSA-ENR), and the control line contains goat anti-mouse IgG. An absorbent pad was used for the absorption of the extra solution, maintaining the flow rate of the solution throughout the strip, and preventing the backflow of the sample (43). Finally, all these components were assembled in order wise on a backing card sheet and cut into 4.0 mm width sections using an AR-100CG cutter, and kept in a desiccator at room temperature for further use.

### Test procedure and principle

**FIGURE 1:**
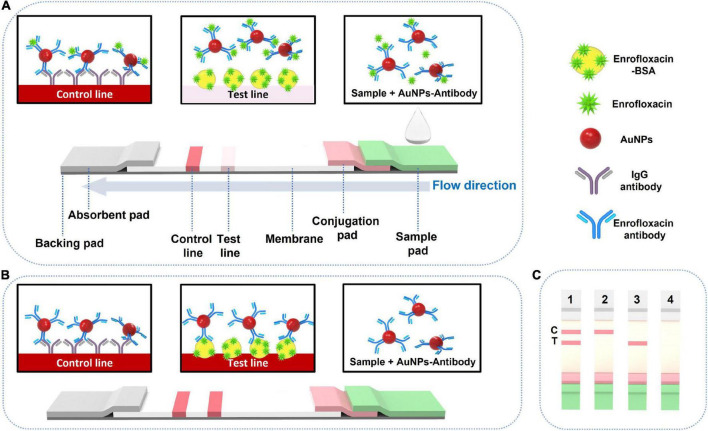
Schematic illustration of LFIA test strip component and working mechanism. T: test line. C: control line. **(A)** Positive testing visual results, **(B)** negative testing visual results, and **(C)** negative visual results (1), positive visual results (2) and invalid results (3 and 4).

The detection principle of LFIA is based on the competitive binding between the target ENR in the samples and coated antigens with the detection probes (44). A 150.0 μl of ENR standard sample solution is added to a 96-well plate, and the LFIA strip is inserted vertically into the well. The sample solution will migrate through the test strip due to capillary force (45) and pass the NC membrane to the absorbent pad. The test results can be seen within 10 min. In case of ENR absence in the sample, the detection reagent that was dried on the conjugation pad (ENR-Ab conjugated AuNPs) will flow through the test pad and will be trapped by the capture reagent (BSA-ENR) to form an easily distinguished test line. On the other hand, if ENR is present in the sample, it competes to bind with the limited amount of detection reagent along with the capture reagent. As the concentration of ENR in the sample is increasing, the test line color intensity will decrease. Consequently, when the sample is containing sufficient ENR, it will fully bind with the detection reagent which in turn blocks the reaction of the detection reagent with the capture reagent by capturing the detection reagent completely. This leads to the invisibility of the T line on the NC membrane which indicates positive results (46). To confirm the validity of the testing procedure of the test strip, the control line should be visible always. In case, color is not appearing on both the T line and C line, or only T line is showing color, these results are likely to be improper or the test strip is invalid, and retesting with a new test strip should be done. A schematic illustration of the LFIA detection principle is shown in Figure 1.

### Sensitivity and specificity of lateral flow immunochromatographic assay strip to enrofloxacin

The sensitivity of the fabricated test strips was measured by detecting ENR in a standard solution. For testing, 150.0 μl of optimized running buffer containing different concentrations of ENR is added to a 96 well plate and dipped the test strip in the solution for 10 min. As the sample solution migrate through the test strip, we could observe two lines indicating the T line and C line. Results were photographed and further analyses were done using ImageJ software. The specificity of the developed test strip was assessed by evaluating the cross-reactivity with other ENR structural analogs (ciprofloxacin, ofloxacin, and norfloxacin), creatine (which is a natural component in meat), and antibiotics (ampicillin, tetracycline, chloramphenicol) used in livestock at a concentration of 50 ng/ml.

### Lateral flow immunochromatographic assay strip testing with spiked milk samples

Milk spiked sample testing was used to estimate the practicability of the strip. Different concentrations of ENR (0, 0.1, 4, 10, 20, 30, 40, 50, and 75 ng/ml) were spiked in milk samples and results were analyzed to evaluate the practicability of the developed test strips.

## Results and discussion

### Evaluation of synthesized colloidal gold nanoparticles

**FIGURE 2:**
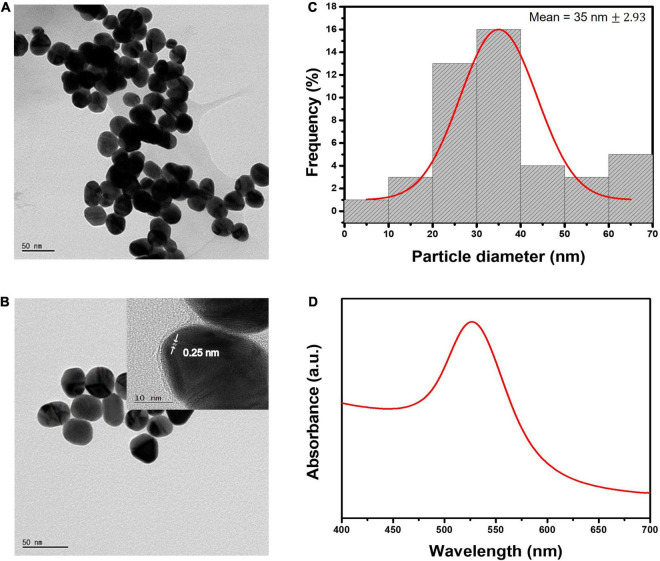
Characterization of AuNPs. **(A,B)** FE-TEM images of AuNPs and the inter-planar spacing. **(C)** Size distribution histogram of 35 nm AuNPs. **(D)** UV-vis absorption spectra of 35 nm gold colloidal solution.

Turkevich-Frens method was used for the growth of AuNPs, where the reducing agent sodium citrate is used as a reductant for HAuCl_4_.3H_2_O (47–49). The approached synthesis method is simple, reproducible, and cost-effective. The shape and size of AuNPs can be manipulated by simply changing the ratio of gold-reductant concentrations (50–52). Obtained AuNPs have good distribution uniformity in the aqueous solution. It is very important to produce consistent reproducible sized AuNPs for biosensor application, as they can affect sensing properties (53). According to literature, the most commonly used AuNPs size varied between 20 and 40 nm, which showed to have the highest sensitivity in LFIA (54). AuNPs with <10 nm size would make them difficult to visually observe the test results. Whereas AuNPs with >40 nm diameter cause instability, and self-coagulation due to steric hindrance during conjugation (55, 56). In this work, the optimized AuNPs size was chosen to be 35 nm. Figures 2A,B depict the FE-TEM images of the synthesized AuNPs with the lattice fringes where the d- spacing of 0.25 nm corresponds to the {111} lattice plane of gold FCC structure (57). Figure 2C shows the size distribution histogram graph of AuNPs with an average diameter of 35 nm ± 2.93. The synthesized AuNPs exhibited absorption spectra peak at 526.7 nm as illustrated in Figure 2D.

### Lateral flow immunochromatographic assay strip optimization

**FIGURE 3:**
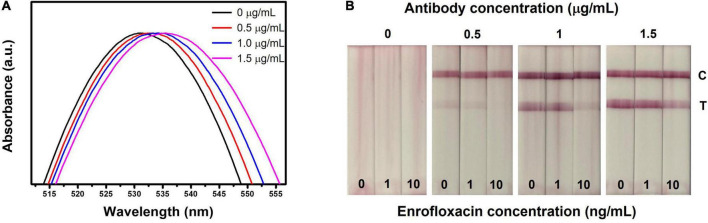
Optimization of LFIA strip for the detection of ENR. **(A)** UV-vis spectrum of various concentrations of ENR-Ab conjugated AuNPs. **(B)** Effect of ENR-Ab with different concentrations (0, 0.5, 1, and 1.5 μg/ml) in LFIA strips results. The ENR sample concentrations are 0, 1, and 10 ng/ml.

The optimization of the LFIA strip is crucial to achieving high sensitivity and specificity (58). Herein, we considered optimizing the parameters (ENR-Ab concentration, blocking buffer, blocking buffer pH, running buffer which includes tween 20 and NaCl). Initially, ENR-Ab with different concentrations (0, 0.5, 1, 1.5 μg/ml) was conjugated with AuNPs and analyzed the respective results. Figure 3A shows the absorption spectra of ENR-Ab conjugated AuNPs. A redshift can be observed in antibody-conjugated AuNPs (0.5, 1, 1.5 μg/ml). AuNPs are affected by molecular interactions and are sensitive to the refractive index of the surrounding medium. Therefore, when the antibody is attached to AuNPs, the refractive index will change, which in turn results in the redshift of the peak (39, 59). Figure 3B portrays the visual results of LFIA test strips with different antibody concentrations (0, 0.5, 1, 1.5 μg/ml), and ENR standard solutions (0, 1, 10 ng/ml). Since there was no significant difference in the redshift with increasing the antibody concentration, we have chosen the optimal antibody concentration based on the visual color intensity of the test line. In Figure 3B, at 0 μg/ml antibody concentration, we didn’t observe any test line or control line on the test strip, but in the presence of antibody, a strong similar color intensity of the control line on the test strip can be seen for the 0, 1, 10 ng/m sample concentrations. This can indicate the successful conjugation of AuNPs and ENR-Ab.

In the presence of various concentrations of antibody, when ENR standard sample solution concentration is 0 ng/ml, a clear visual test line can be observed indicating the absence of ENR. On the other hand, in the presence of ENR, the test line color intensity is decreasing with increasing the ENR sample concentration. It should be noted that the test line is disappearing at antibody concentrations of 0.5 and 1 μg/ml and sample concentrations of 10 ng/ml. This indicates that the ENR concentration in the sample is the same or above the detection limit indicating that all the AuNPs conjugated Ab (AuNPs-Ab) probes have bounded to ENR in the test sample and thus leaving no available probes to bind with BSA-ENR on the test line, resulting in no color formation at the test line. However, if the antibody concentration is exceeding the detection limit, this will leave free AuNPs-Ab probes to bind at the test line, resulting in decreased sensitivity of the test strip as shown in Figure 3B where the concentration is 1.5 μg/ml and 10 ng/ml for antibody and sample concentration, respectively. Therefore, detection limit and sensitivity are affected by antibody concentration (60). In this work, the optimal antibody concentration was set to be 1 μg/ml, which is considered to be sufficient for visual detection.

Secondly, blocking buffer and running buffer conditions were optimized. Blocking buffer and running buffer will show a strong influence on the sensitivity and specificity of LFIA as they minimize the non-specific binding interactions between molecules and with the test strip (61, 62). Herein, 10 mM PB, 10 mM PBS, and 20 mM borate buffer with pH 9.0 were used for optimizing the blocking buffers. Supplementary Figure 1 shows the comparison between the buffers and their effect on the detection results with 0, 0.1, 1, and 10 ng/ml ENR sample solution concentrations. From Supplementary Figure 1 it is evident that 20 mM borate buffer showed the best visual results as test line color intensity and distribution were superior, so borate buffer was chosen as the optimal buffer for further experiments. Further, experiments were carried out to optimize the various blocking buffer pH (7, 8 and 9) values of the 20 mM borate buffer. From Supplementary Figure 2 it is evident that 20 mM borate buffer with pH value 9 has shown the best visual results as test line color intensity and distribution were superior. So, 20 mM borate buffer with pH 9 was chosen for better visual detection. Further, Tween 20 and NaCl in the running buffer were optimized. Supplementary Figure 3 shows the optimization results of Tween 20 with different concentrations (0, 2, 4, 5, 6, 8, and 10 %). Tween 20 is a surfactant that is widely used to optimize the flow rate of the sample through test strips (63). From Supplementary Figure 3 it is evident that a 5% concentration of Tween 20 in the running buffer has shown appreciable visual results with the least background effect. Finally, optimization experiments of NaCl in the running buffer were performed. Supplementary Figure 4 shows the optimization results of NaCl in running buffer with various concentrations (0, 18.75, 37.5, 75, 150, and 300 %). With increasing the concentration of NaCl, the test line color intensity is increasing. However, for 300 mM NaCl the AuNPs-Ab conjugate did not fully flow through the test strip and stuck at the edges of the conjugation pad. Significantly, 150 mM NaCl concentration has shown clean background with a strong test line signal. Therefore, 150 mM NaCl concentration has been chosen as the optimized concentration.

### Lateral flow immunochromatographic assay test strips sensitivity in standard solutions

**FIGURE 4:**
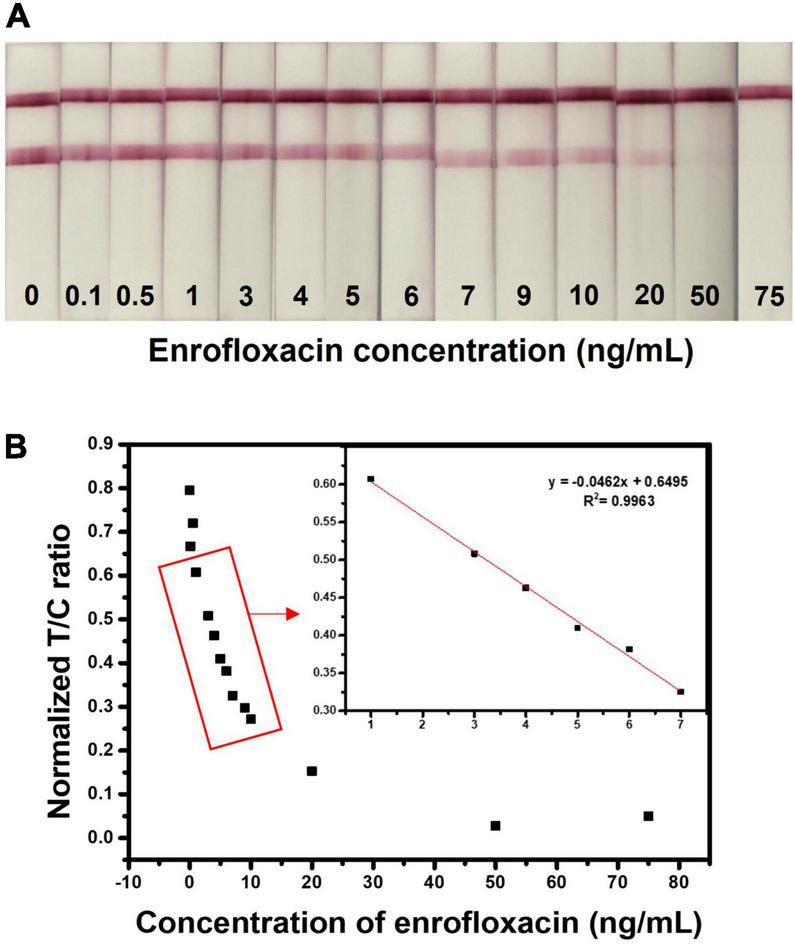
Detection of ENR in standard solution. **(A)** Assessment of sensitivity of the developed test strip by detecting various concentrations of ENR (0, 0.1, 0.5, 1, 3, 4, 5, 6, 7, 9, 10, 20, 50, and 75 ng/ml) standard solutions in the borate buffer. **(B)** Relationship between normalized T/C ratio and the ENR concentration. The normalized T/C ratio was calculated by removing the background value of test strips by the ImageJ program.

The LFIA test strip was optimized by assessing different antibody concentrations, buffers, buffer pH, blocking buffer, and running buffer concentrations and optimized conditions were used for the evaluation of test strip sensitivity towards ENR. The sensitivity of the developed test strip was assessed by the detection of different concentrations of ENR (0, 0.1, 0.5, 1, 3, 4, 5, 6, 7, 9, 10, 20, 50, and 75 ng/ml) standard solutions in the borate buffer. For semi-quantitative analysis, the visual limit of detection (vLOD) was determined as the minimum concentration of ENR that results in a weaker color on the test line than that of the negative sample. Whereas the threshold concentration of ENR that results in the complete disappearance of the test line is the cut-off value (64). As shown in Figure 4A, the test line is slowly disappearing with increasing the ENR concentration. Therefore, the observed vLOD for the LFIA test strip was 20 ng/mm and the cut-off value was 50 ng/ml with a visual detection range between 0 and 20 ng/ml. The proposed test results can be visually examined within 10 min. Figure 4B illustrates the analysis of ENR test strips using the ImageJ program. The T/C ratio was calculated by test line intensity over control line intensity. The normalized T/C ratio was calculated by removing the background value of the test strips. The LFIA test strips achieved a dynamic range of 1-75 ng/ml with the linearity of *R*^2^ = 0.9963. These results indicate the rapid and good sensitivity of the developed sensor.

### Lateral flow immunochromatographic assay test strips specificity for enrofloxacin

**FIGURE 5:**
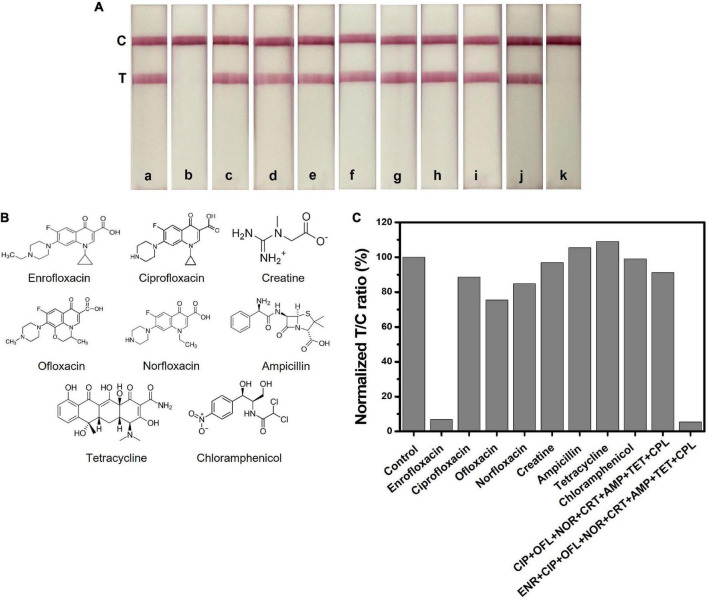
Selectivity test of ENR and interference molecules at a concentration of 50 ng/ml. **(A)** Images of the LFIA test strips with ENR and interference molecules. a: control, b: enrofloxacin (ENR), c: ciprofloxacin (CIP), d: ofloxacin (OFL), e: norfloxacin (NOR), f: creatine, g: ampicillin (AMP), h: tetracycline (TET), i: chloramphenicol (CPL), j: mixture of CIP+ OFL+ NOR+ CRT+ AMP+ TET+ CPL and k: mixture of ENR+ CIP+ OFL+ NOR + CRT+ AMP+ TET+ CPL. **(B)** Chemical structures of ENR and other interference molecules. **(C)** The normalized T/C ratio of LFIA strips produced by the ImageJ program.

Specificity evaluation of the LFIA test strip was confirmed through a cross-reactivity test. We tested the LFIA test strip with possible interference molecules such as ENR analogs (ciprofloxacin (CIP), ofloxacin (OFL), norfloxacin and (NOR)), creatine (CRT), and commonly used livestock antibiotics such as (ampicillin (AMP), tetracycline (TET), and chloramphenicol (CPL)) at a concentration of 50 ng/ml. Figure 5A demonstrates the image of the test strips that were tested for cross-reactivity. Initially, each compound was tested separately and later tested as a mixture to confirm the antibody specificity. As we can see, the test line is only disappearing when ENR is present in the sample, and all test lines other than ENR are showing an evident red test line. Figure 5B depicts the structures of the interference molecules. The test line color intensity was analyzed through the ImageJ program and the graph is shown in Figure 5C. Most of the compounds show similar results to the negative sample. Therefore, the obtained results showed high specificity of the LFIA test strip towards ENR sensing, which makes it a suitable sensor for detecting ENR in foods.

### Enrofloxacin spiked milk samples analysis

**FIGURE 6:**
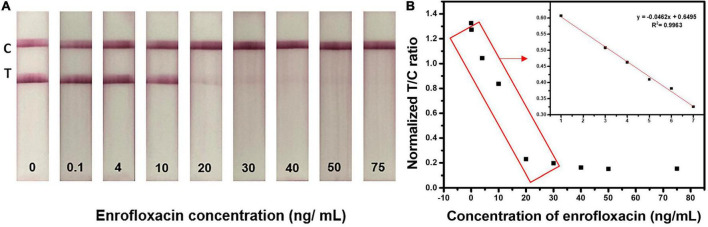
Detection of ENR in milk samples using optimized LFIA strips. Images of the LFIA test strips for detecting ENR in milk samples with 0, 0.1, 4, 10, 20, 30, 40, 50, and 75 ng/ml concentrations.

To analyze the performance of the developed LFIA sensor for ENR detection and screening in real samples, we tested the ability of the fabricated LFIA in detecting the ENR in the spiked milk sample. The purchased real milk sample did not have any pretreatment steps to ease the test procedure. Different concentrations of ENR (0, 0.1, 4, 10, 20, 30, 40, 50, and 75 ng/ml) were spiked in milk samples and analyzed. Figure 6 shows the strip test results after 10 min reaction with spiked milk samples. The obtained vLOD is 20 ng/ml with a cut-off value of 50 ng/ml. Similarly, the normalized T/C ratio was calculated by removing the background value of test strips and the results are shown in Supplementary Figure 5. The T/C ratio was calculated by test line intensity over control line intensity. Test line and control line intensity was measured using the ImageJ program. The test strips showed a dynamic range of 0.1-30 ng/ml with the linearity of *R*^2^ = 0.9266. These results evidence that the developed ENR test strip can sufficiently detect ENR in milk samples with appreciable sensitivity as shown in ENR standard solution. The test strips are suitable for the detection of ENR at MRL (50 ng/ml) or lower which can provide an idea of too high or too low concentration of ENR in milk sample making it very useful as qualitative analysis to identify concentrations that are above or below MRL (65).

## Conclusion

In summary, a rapid and sensitive LFIA sensor was fabricated with simple testing procedures for the detection of ENR using AuNPs conjugated antibody. For sensitivity improvement, many optimization steps were conducted on LFIA test strips. The developed LFIA sensor was applied for the detection of ENR in milk samples in a single step without sample pretreatment. The vLOD was found to be 20 ng/ml with a cut-off value of 50 ng/ml in the milk samples. The obtained LOD value of the test strip was within the safety limit guidelines of MFDS, South Korea. The test strip showed negligible cross-reactivity with ENR analogs, and other interference molecules, this indicates the high specificity of the LFIA test strip towards ENR. The designed test strip showed good reliability. The visual test results can be seen within 10 min without the need for special equipment. The fabricated sensor is suitable for the rapid and on-site detection of ENR in milk samples. Therefore, the test strip can be employed as a potential detection strategy for the qualitative on-site detection of ENR.

## Data availability statement

The original contributions presented in this study are included in the article/Supplementary material, further inquiries can be directed to the corresponding authors.

## Author contributions

MA: methodology, data curation, validation, investigation, and writing – original draft. JY: methodology. SS: review and editing. SP: analysis. RU and M-HO: conceptualization, formal analysis, and writing – review and editing. YH: conceptualization, project administration, investigation, and writing – review and editing. All authors contributed to the article and approved the submitted version.
