# Supplementary material for: A highly sensitive lateral flow immunoassay for the rapid and on-site detection of enrofloxacin in milk
Source: Front Nutr. 2022 Oct 24;9:1036826. doi: 10.3389/fnut.2022.1036826 (PMC9637957; doi:10.3389/fnut.2022.1036826)
Supplement: Supplementary file 1 [file Table_1.DOCX]

Supplementary Material


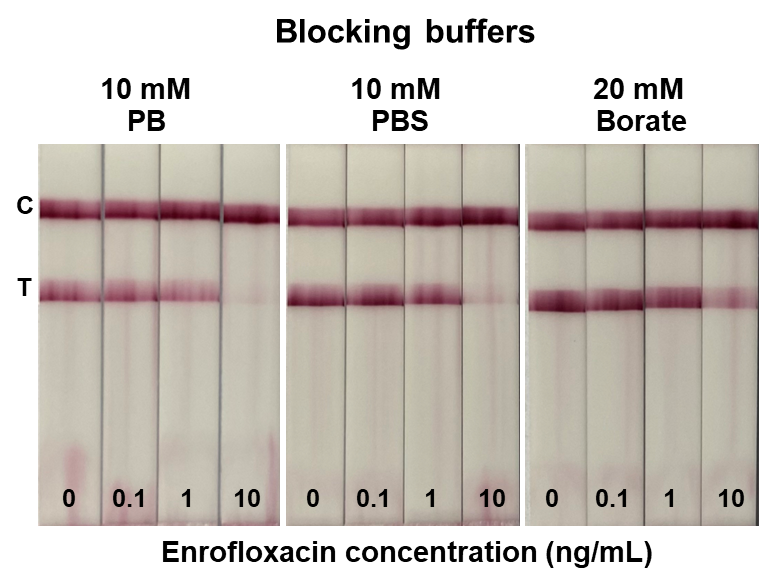


**Supplementary Figure 1.** Optimization of LFIA strip for the detection of enrofloxacin. Effect of the various types of blocking buffers on LFIA strips. The enrofloxacin sample concentrations are 0, 1, and 10 ng/mL.

**
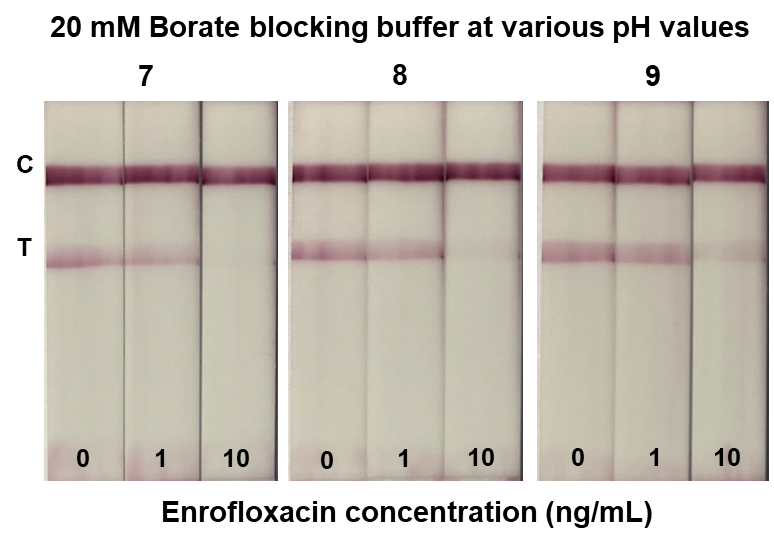
**

**Supplementary Figure 2.** Optimization of LFIA strip for the detection of enrofloxacin. Effect of the various pH values (7, 8, 9) of the 20 mM borate buffer on LFIA strips. The enrofloxacin sample concentrations are 0, 1, and 10 ng/mL.

**
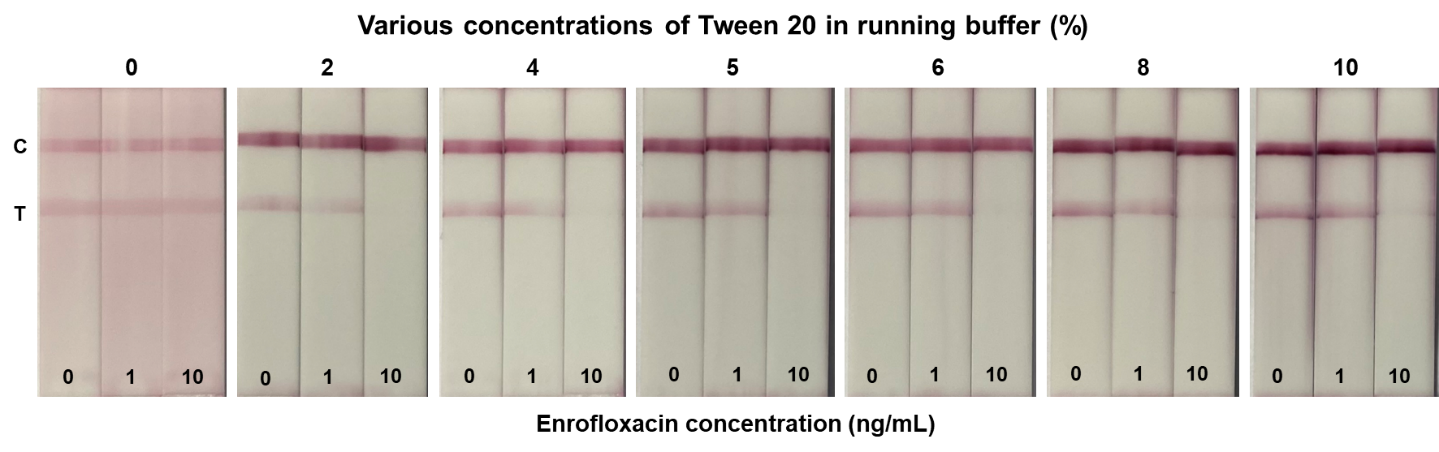
**

**Supplementary Figure 3.** Optimization results of Tween 20 buffer with different concentrations (0, 2, 4, 5, 6, 8, and 10 %) on LFIA test strips. The enrofloxacin sample concentrations are 0, 1, and 10 ng/ mL.


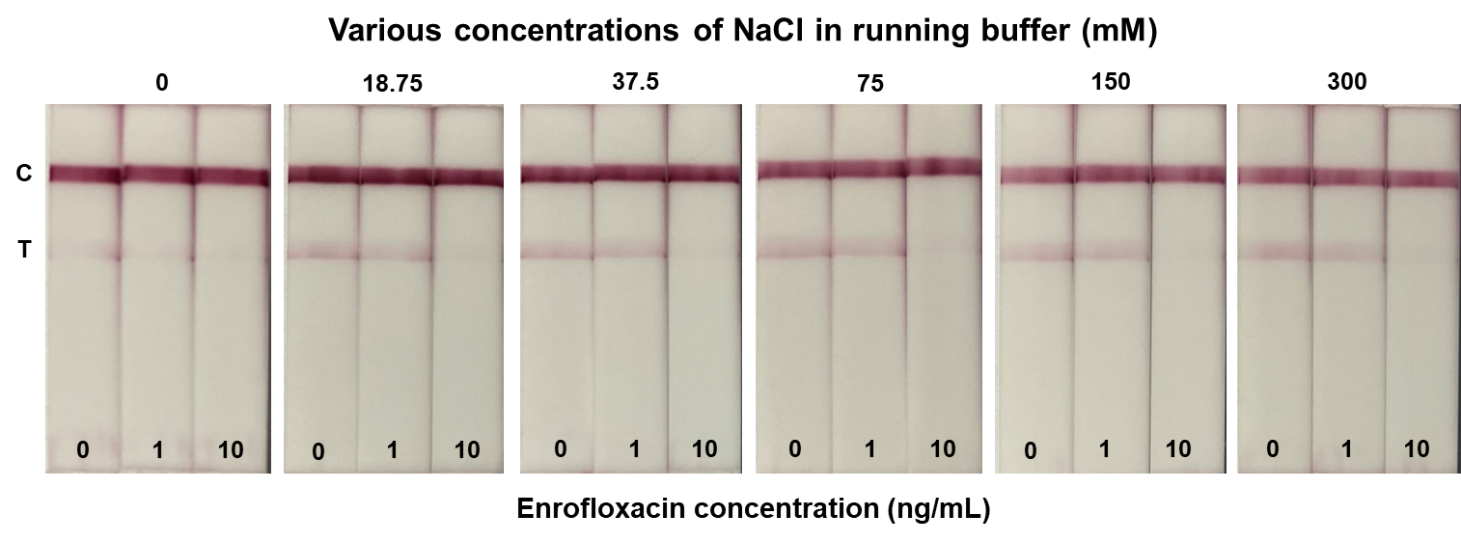


**Supplementary Figure 4.** Optimization results of NaCl buffer with different concentrations (0, 18.75, 37.5, 75, 150, and 300 mM) on LFIA test strips. The enrofloxacin sample concentrations are 0, 1, and 10 ng/ mL.


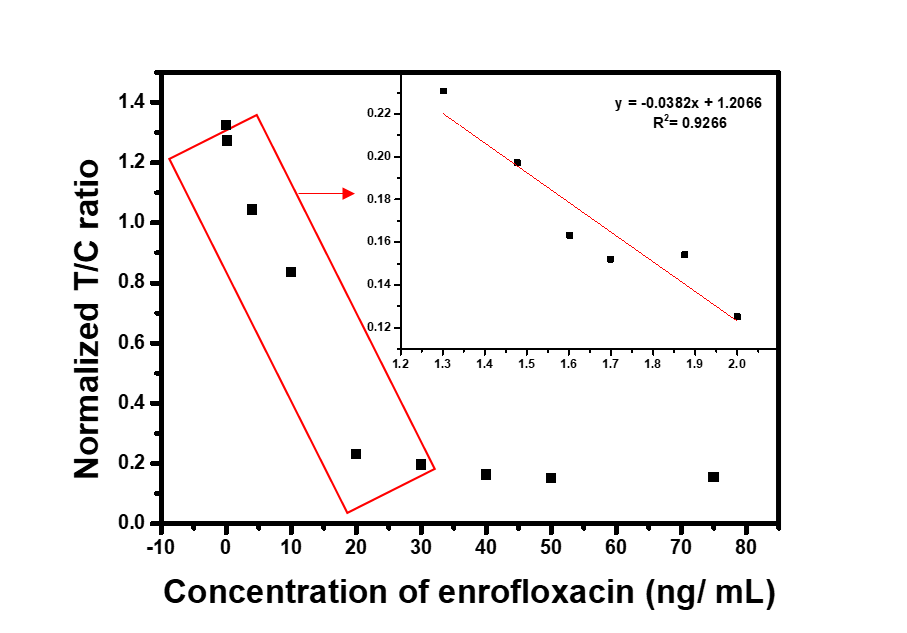


**Supplementary Figure 5.** Real-time analysis. Relationship between normalized T/C ratio and the enrofloxacin concentration in milk samples. The T/C ratio was calculated by test line intensity over control line intensity. Test line and control line intensity was measured using the ImageJ program.
